# The 2016 Basal Ganglia Gordon Research Conference and Gordon Research Seminar

**DOI:** 10.1038/npjparkd.2016.17

**Published:** 2016-07-14

**Authors:** Harry S Xenias, Mark D Bevan

**Affiliations:** 1 Department of Physiology, Feinberg School of Medicine, Northwestern University, Chicago, IL, USA

## Abstract

The second bi-annual Basal Ganglia Gordon Research Conference (GRC) was held 28 February through 4 March 2016 in Ventura, California, USA. Two hundred attendees participated and 46 lectures and 127 posters were presented. The meeting also incorporated a Gordon Research Seminar (GRS), held between February 27–28, organized exclusively by and for graduate and post-doctoral researchers. The GRC and GRS attracted scientists from across the globe with 4 continents and 18 countries represented. Research was presented in oral and poster formats during themed sessions. Lecturers ranged from graduate and post-doctoral trainees to junior and more established principal investigators. Both basic and clinician scientists were also well represented. The latest basal ganglia research discoveries on both normal function and diseases, such as Parkinson’s disease (PD), Huntington’s disease (HD), addiction and compulsive disorders, were communicated. In addition, government, industry, and clinical leaders delivered an optional Translational Café that focused on therapy development. In summary, the 2016 Basal Ganglia GRC and GRS showcased state-of-the-art research, promoted discussion, and interaction throughout the research community, and most likely inspired the next wave of fundamental and translational scientific discoveries in this brain region.

Gordon Research Conferences (GRC) cover a diverse range of subjects in the physical, chemical, and biological sciences. Despite the breadth of topics the GRC has covered during its 70-year existence, it was not until 2014 that a GRC was created for the basal ganglia. The basal ganglia are a network of interconnected subcortical brain nuclei that are important for movement, association, and motivation, and are involved in psychomotor disorders like Parkinson’s disease (PD), Huntington’s disease (HD), addiction, and obsessive-compulsive disorder. The creation of the Basal Ganglia GRC has come at a time of extraordinary technological advances in neuroscience. New research tools include: rapid, large-scale genome, and proteome sampling; highly efficient, targeted viral vectors; genetically encoded calcium and voltage indicators; *in vivo* fiber photometry and endomicroscopy; 2-photon and super-resolution microscopy; optogenetics and chemogenetics. Together, these approaches have allowed unprecedented cell class- and circuit-specific interrogation and manipulation of the basal ganglia, yielding deep insight into their operation in health and disease. The conference title, ‘Emerging Views of Cellular and Circuit Diversity Within the Basal Ganglia,’ reflects the impact that these new research tools have made on the field’s research.

Drs Nicole Calakos (Duke University) and Mark Bevan (Northwestern University) served as chair and vice-chairs, respectively, of this year’s conference, attended by 200 scientists (Figure 1). Dr D James Surmeier, chair of the inaugural 2014 Basal Ganglia GRC, opened the 2016 conference (https://www.grc.org/programs.aspx?id=16708) with an evening lecture on ‘Striatal Adaptations in PD: When Homeostasis Goes Awry.’ The format for the next 4 days were morning and evening sessions each consisting of 5–7, 10–20 min talks with 5–10 min discussion after each talk. There were 8 sessions on a diverse range of topics including: circuit basis of behavior; computation; habit, compulsion, and addiction; dysfunction in neurodegenerative disease; cellular and molecular diversity; action selection; synaptic plasticity mechanisms, and signal transduction. Of the 46 lectures, 16 were selected competitively on the basis of submitted abstracts. These talks were shorter and generally delivered by junior scientists. The consensus was that these were a particular highlight of the meeting. This year, poster presenters ‘advertised’ their work through creative, fifty-word oral ‘tweets’ at the end of each morning session. Posters were then presented that afternoon between 16:00  and 18:00  hours. A defining feature of this meeting (and all other GRCs), in contrast to other scientific meetings, was the opportunity for informal interaction, networking, and collaboration building, through communal dining, informal gatherings, and afternoon free-time periods when attendees take part in organized outings or other activities.

A prominently covered topic throughout the meeting was basal ganglia dysfunction in PD and HD. The molecular, cellular, and circuit mechanisms underlying these diseases were addressed in multiple talks employing cutting-edge approaches in animal models but the human burden of these diseases was highlighted by clinician presentations with patient footage. Another fascinating topic was devoted to the cortico-basal ganglia-thalamo-cortical circuits underlying habit, compulsion, and addiction led by Dr Christian Lüscher, University of Geneva. Here progress identifying the circuits and mechanisms underlying this spectrum of adaptive and less adaptive behaviors was reported. Another stimulating session was devoted to our emerging appreciation of the molecular and cellular diversity of the basal ganglia, led by Dr Bernado Sabatini, Harvard Medical School/Howard Hughes Medical Institute. Here the somewhat daunting diversity of basal ganglia neurons from molecular, anatomical, physiological, and functional perspectives was detailed. Other sessions were devoted to the role of the basal ganglia in action selection, movement, and cognitive processing, and to the computational principles underlying these diverse functions. The implications of synaptic plasticity mechanisms for behavior and signal transduction mechanisms at the cellular and molecular levels concluded the formal sessions. An optional Translational Café was also held, in which funding, industry, and clinical leaders discussed with attendees the translational process from funding mechanisms, to preclinical research, to clinical trials, and the administration of novel therapeutics to patients.

Another notable addition since the 2014 conference was the first Basal Ganglia Gordon Research Seminar (GRS) hosted by Dr Julia Lemos (National Institutes of Health (NIH)) and Justin O’Hare (Duke University). This one and a half day event preceded the start of the GRC and was entirely organized and presented by graduate and postdoctoral scientists. The GRS possessed the essential elements of the GRC, including session talks, post-talk discussions, and poster sessions. The Keynote lecture was given by Dr Talia Lerner, Stanford University on ‘Intact-Brain Analyses Reveal Distinct Information Carried by SNc Dopamine Subcircuits’. There were also oral sessions entitled ‘Novel Methods for Measuring Cellular and Circuit Dynamics of the Basal Ganglia’ and ‘From Bench to Bedside: Focus on Understanding the Role of the Subthalamic Nucleus in Basal Ganglia Function and Dysfunction.’ The GRS ended with a panel discussion on career development and job opportunities, entitled ‘Deciding Which Career Track in Science Is the One for You.’ Roger Cachope (CHDI Foundation, USA), Aryn Gittis (Carnegie Mellon University), Meghan Mott (National Institute of Neurological Disorders and Stroke, NIH), and Sebastien Thuault (Nature Neuroscience) each spoke about their backgrounds and the factors that had informed their career choices.

In summary, the 2016 Basal Ganglia GRC and associated GRS showcased advances in a wide variety of research areas and together provided a key forum for the research community to present new discoveries and discuss their significance, and to frame the next set of critical scientific questions to be addressed. After the conclusion of the conference, one could not help feel both inspired and excited for future progress. Indeed, we predict that the rate of impactful discovery will accelerate and in our lifetimes we will largely understand the multifaceted nature of basal ganglia function, and be more able to effectively treat or prevent basal ganglia diseases.

## Figures and Tables

**Figure 1:**
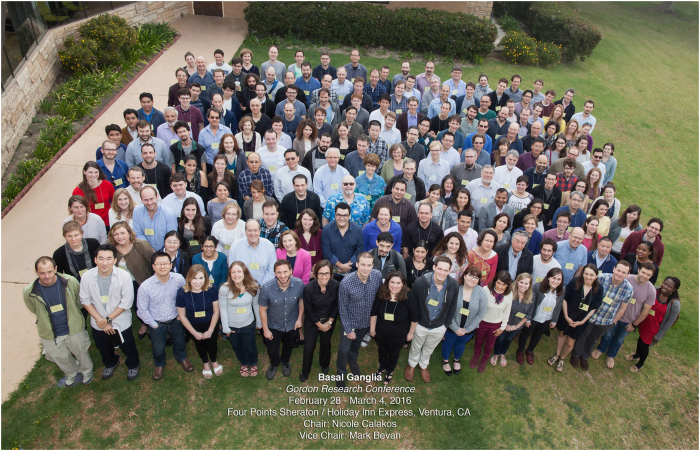
Group photograph of the attendees of the 2016 Basal Ganglia Gordon Research Conference and Gordon Research Seminar.

